# Improved DC Dielectric Performance of Photon-Initiated Crosslinking Polyethylene with TMPTMA Auxiliary Agent

**DOI:** 10.3390/ma12213540

**Published:** 2019-10-29

**Authors:** Peng Qiu, Jun-Qi Chen, Wei-Feng Sun, Hong Zhao

**Affiliations:** Key Laboratory of Engineering Dielectrics and Its Application, Ministry of Education, School of Electrical and Electronic Engineering, Harbin University of Science and Technology, Harbin 150080, Chinachen89670420@163.com (J.-Q.C.); sunweifeng@hrbust.edu.cn (W.-F.S.)

**Keywords:** crosslinking reaction, ultraviolet irradiation, space-charge accumulation, electrical conductance, first-principles calculation

## Abstract

To achieve high direct current (DC) dielectric performance of crosslinked polyethylene (XLPE) applied for insulated cable, the auxiliary crosslinking agent of trimethylolpropane trimethacrylate (TMPTMA) is employed in photon-initiated crosslinking process to the present polar-molecular group which will introduce deep traps for charge carriers. The space-charge accumulation and electrical conductance of XLPE are observably suppressed due to the deep traps deriving from the TMPTMA crosslinkers that are chemically connecting (grafted onto) polyethylene molecules. Thermally stimulated depolarization current tests and first-principles calculations consistently demonstrate a trapping mechanism of impeding charge injection and carrier transport in XLPE with TMPTMA crosslinkers. The characteristic cyclic anhydrides with coupled carbonyl groups are used as auxiliary crosslinkers to promote crosslinking efficiency and provide polar groups to polyethylene molecules which can be effectively fulfilled in industrial cable production. The results of infrared spectroscopy show that the auxiliary crosslinkers have been successfully grated to polyethylene molecules through the UV-initiation process. The space-charge characteristics achieve a significant improvement consistent with the theoretical estimation that deeper electronic traps can be introduced by auxiliary crosslinker and will consequently suppress space-charge accumulation through a trapping mechanism. Meanwhile, the conductivity of XLPE observably increases after using TMPTMA auxiliary crosslinkers at various temperatures of cable operation. The first-principles calculations also demonstrate that substantial electronic bound states have been introduced at the band edge of polyethylene molecules crosslinked by TMPTMA, leading to reduction in electrical conductivity. On the advantage of ameliorating DC dielectric performance by way of UV-initiated crosslinking process, the present research suggests a substantial strategy in XLPE cable industrial productions.

## 1. Introduction

At present, the effective methods of modifying polymer insulation materials have been presented as by polymer dielectric nanocomposites (filling nanoparticles), ultra-clean process, blending, and chemical modification [1,2,3]. The underlying modification mechanism and realizing their applications in the industrial production of insulated cables have always been advanced topics. It was especially noted that the dielectric performance of insulated polymers can be evidently modified by filling nanoparticles. However, the space-charge accumulation and electrical conductance of nanocomposites depends greatly on the contact area between nanofillers and polymeric matrix, which is required to control the nanofillers in minimal size and high dispersivity [4]. Furthermore, high concentration of inorganic nanofillers to obtain nonlinear composites generally used in insulation systems will inevitably deteriorate mechanical properties and break down strength. These drawbacks and difficulties in developing cable accessories by nanodielectrics technology make it almost impossible to be fulfilled in practical industrial productions. Employing the first-principles calculations, Meunier studied the energetic feature of intrinsic traps introduced by physical and chemical defects and reasonably suggested that the polar group can present deep traps in polymer materials [5,6]. Recent reports indicated that excellent dielectric properties of modified polymers by chemically grafting are attributed to the trapping mechanism of space-charge suppression and break down strength improvement [7]. However, due to low gasification temperature, the polar molecules are liable to vaporize in chemical grafting reactions so as to form gas bubbles in the pipeline of crosslinked polyethylene fabrication, which will cause severe degradation on insulation performance. Moreover, it is inevitable to produce by-product impurity in chemical grafting reactions, which need to be carried out at high temperature and pressure for a long time, leading to mechanical deterioration and conductivity augmentation of polymer materials. Therefore, the traditional chemical method of grafting micro-molecules cannot be feasible for industrial cable manufactures.

With the development of the marine development strategy, the submarine cable manufacture of high-grade and super-long crosslinked polyethylene (XLPE) has become a focus in electrical insulation research. However, the peroxide chemical crosslinking technology commonly used in the production of high-voltage cables represents some unavoidable shortcomings, such as high production energy consumption, low heat-transfer rate, long reaction time, and so on. In addition, scorching is liable to occur at the dead angle of mold in chemical crosslinking processes, thus forming defects in the insulation layer of high-voltage cable, which will worsen electrical characteristics [8,9]. Ultraviolet (UV) crosslinking technology is a characteristic non-thermosensitive grafting method with high production efficiency. A series of unique advantages, such as fast and continuous long time processing, small capital investment, and low consumptions of raw materials and energy in cable manufactures, make the UV-initiation technique stand out in crosslinking technology, and are promised to realize high voltage grade and super-length crosslinked cable in practical industrial productions [10,11,12,13]. The crosslinking configurations of polyamides and rubbers can be modified by employing triallyl cyanurate (TAC), Triallyl isocyanurate (TAIC), and trimethylolpropane trimethacrylate (TMPTMA) compounds with functional groups as auxiliary crosslinking agents in the polymer crosslinking process, resulting in considerable improvements of mechanical properties and thermal stability [14,15]. This strategy can also be exploited to improve the crosslinking degree of polyethylene, which, however, is rarely reported. The crosslinking configuration of UV-initiated XLPE is related to the double-bond conversion and polymerization degree of auxiliary crosslinker monomers, leading to bridged crosslinking networks between polyethylene molecules [16]. Our research team has studied the crosslinking mechanisms of photon-auxiliary crosslinking agents such as TAIC, N.N-m-phenylene dimaleimide, and TMPTMA in UV-initiated polyethylene crosslinking process, and investigated their effects on DC dielectric properties of XLPE, as it has been previously reported that TAIC can remarkably quicken the crosslinking rate from minutes to seconds [17]. We also found that TMPTMA represents the highest efficiency to improve DC dielectric performance of XLPE, while there have so far been no reports on exploiting TMPTMA in UV-initiated crosslinking process for ameliorating dielectric properties of XLPE.

In the present paper, to develop the prospective molecular modification of polymer insulation materials, a strategic scheme of UV-initiated crosslinking reactions between the polyethylene molecules and the auxiliary crosslinking agent is employed to achieve XLPE materials with preferable dielectric performance, which can be practically used in industrial cable manufacture. We adopt TMPTMA with triple-carbonyl polar groups as an auxiliary crosslinking agent and benzophenone (BP) as the photon-initiator to accomplish crosslinking reactions of polyethylene molecules under UV irradiation, which will simultaneously introduce deep traps for charge carriers to ameliorate direct current (DC) dielectric properties. Linear low-density polyethylene (LLDPE) represents higher resistances to penetration, impact, and tear than ordinary low-density polyethylene, which makes it suitable to be processed into thin films. In particular, LLDPE is adapted to advanced plastic processes, such as film extrusion process, due to its excellent melting rheological property with a melt rheological index of 1.0 g/10 min. Furthermore, LLDPE shows remarkable endurance to environmental stress cracking, low temperature impact, and mechanical warping, which can be effectively applied in the extrusion and molding of pipe and sheet substances. Without long-chain branches on the molecular backbone, LLDPE molecules can easily form crosslinking and be grafted by functional molecular groups in crosslinking reactions, which facilitates the developments of a new crosslinking process and graft-modified polyethylene materials. Therefore, LLDPE is used as the pristine matrix material for preparing the modified XLPE materials in the present study. The first-principles electronic structure calculations are performed to consistently elucidate the electronic bound states with the energy level located in band-gap arising after polyethylene molecules are chemically connected by the auxiliary crosslinker of TMPTMA, which accounts for the underlying mechanism of inhibiting space-charge accumulation and reducing electrical conductivity.

## 2. Experiments and Theoretical Schemes

### 2.1. Material Preparation

The melting blend and hot-pressing approaches are employed in synthesis of TMPTMA-crosslinked polyethylene with the original materials being adopted as follows: linear low-density polyethylene (LLDPE, DFDA-7042, Sinopec Company Ltd., Beijing, China) as parent material, benzophenone (BP, Jinleiyuan Chemical Co., Ltd., Lianyungang, China) for initiating grafting reaction under photon irradiation, auxiliary crosslinking agent (TMPTMA, Sinopharm Chemical Reagent Co., Ltd., Shanghai, China), all of which have purity higher than 95%. In the melting blend process of preparing the initial mixtures, the pristine LLDPE are melted uniformly in Torque Rheometer (RM200C, Hapro Company Ltd., Harbin, China) at 160 °C for 3 min with stirring speed of 60 rpm, and then 2 wt. % BP photon-initiator and 1 wt. % auxiliary crosslinking agent are added and blended for 3 min then cooled to room temperature to obtain the uniform mixture materials. 

**Figure 1:**
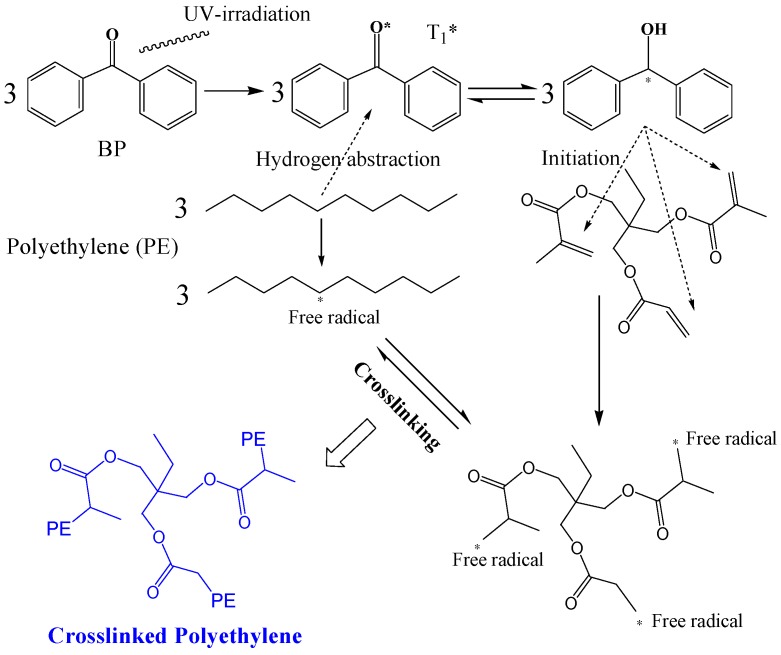
Schematic illustration of UV-initiated crosslinking reactions employing TMPTMA as auxiliary crosslinking agent.

For photon-initiated crosslinking reactions, the prepared hot-pressed blend is first treated in a plate vulcanizer at 160 °C with the pressure being increased by 5 MPa per 5 min from 0 to 15 MPa so as to make the material melt, and then the melt material is irradiated by a light source array of UV LED units (NVSU233A-U365, Riya Electronics Chemistry Co., Ltd., Shanghai, China) for 2 s on an irradiation platform at normal pressure and room temperature in an air atmosphere. In this pivotal process, exploiting UV-transparency of the LLDPE fluid at the temperature higher than fusion point after being squeezed out from extruder, the UV lights are incident through the melting mixture of LLDPE, BP, and TMPTMA. Hydrogen abstractions from polyethylene molecules to UV-excited first-triplet (T_1_) BP and the partial fractures of carbon double bonds on TMPTMA molecules are simultaneously initiated by instantaneous UV irradiation, leading to considerable amounts of transient free radicals on both polyethylene and TMPTMA molecules which will eventually form chemical bonds to accomplish crosslinking reactions, as schematically illustrated in Figure 1. Since they are assisted by TMPTMA, the free radicals on polyethylene molecules connect to each other though TMPTMA and thus form a reticulated crosslinking system which is nominated here as XLPE-TMPTMA. The UV-initiated XLPE with TMPTMA crosslinker (UV-XLPE-TMPTMA) materials are finally achieved after short-circuit degassing at 80 °C for 48 h in a vacuum oven to eliminate the residual impurities of small molecules. In photon-initiated crosslinking process under UV irradiation, the power and wavelength of light-emitting are controlled on 1.0 W and 365 nm respectively, and light incident direction is 60° angle with the plane of thin film sample. Moreover, to ensure homogeneous crosslinking reaction across laterally film materials, the samples are mounted on the conveyor belt at a constant speed of 1.5 mm/s with a distance of 15 mm between the film plane and UV source.

### 2.2. Characterization and Measurement

To detect the molecules of auxiliary crosslinking agent being grafted onto polyethylene molecular chains through UV-initiated crosslinking reactions, the molecular structures of prepared samples are characterized by Fourier Transform Infrared (FT-IR) Spectroscopy (FT/IR-6100, Jiasco Trading Co., Ltd., Shenyang, China) in spectral range of 500 ~ 4000 cm^−1^ with a scanning resolution of 2 cm^−1^. Complying to the standards of GB/T 2951.21-2008 and ASTM D 2765-2011 respectively, the crosslinking degree of XLPE is tested by thermal elongation and gel extraction experiments in which the prepared materials are pressed into dumbbell-shaped samples under 0.2 MPa and then degassed for 30 min at 200 °C in vacuum oven.

Using a pulsed electro-acoustic system (HY-PEA-DPT01, HeYi Electric Co., Ltd., Shanghai, China), the space-charge distributions are measured at ambient temperature of 25 °C by applying an electric field of 40 kV/mm in polarization for 30 min and then short-circuiting for 30 min, in which the tested materials are fabricated into 50 × 50 × 0.3 mm^3^ film samples with both sides being evaporated by aluminum electrode films in 25 mm diameter. Thermally stimulated depolarization currents (TSDC, Harbin University of Science and Technology, Harbin, China) are tested to analyze the energy level distribution of charge traps, for which the samples in 0.1 mm thickness are first polarized under an electric field of 40 kV/mm for 30 min and then rapidly cooled by liquid nitrogen down to −50 °C being stabilized for 3 min. The depolarization currents are measured in short circuit when temperature is raised from −50 to 150 °C with a heating rate of 3 °C/min.

Electrical conductivity is measured by a three-electrode system at variable temperatures from 25 to 90 °C for the circular film samples of 50 mm diameter and 300 µm thickness with the evaporated aluminum electrodes on both sides. The protective electrode in annular shape (inner and outer diameters of 54 mm and 76 mm, respectively) encircles the disc of the measuring electrode (50 mm in diameter) on one side of the film samples, and the circular electrode with a larger diameter of 78 mm on the other side is used for applying high voltage. After the samples are heated and stabilized at the testing temperature for 60 min, the electric field at each point covering the range of 3–40 kV/mm is applied for 60 min to measure the stable conductance current.

### 2.3. Molecular Model and Theoretical Methodology

The model of polyethylene molecules in 20 polymerization degrees, being connected by an auxiliary crosslinker molecule near the middle position of polyethylene backbone chain, is initially constructed with random distributed torsion based on rotational isomeric state (RIS) model [18]. The constructed initial polymer configurations are geometrically optimized to structural relaxation by total energy functional minimization with conjugated gradient algorithm in first-principles calculations [19], so that the energy change, atomic force, and displacement are theoretically evaluated to be lower than 1.0 × 10^−5^ eV/atom, 0.03 eV/Å and 0.001 Å, respectively. The electronic structures are calculated for molecular orbitals and electronic density of states to investigate band-edge features and grafting-introduced trap states. The first-principles calculations are performed by employing the scheme of all-electron numerical atom-orbitals as implemented in DMol3 program of Materials studio 8.0 software package (Accelrys Inc., Materials Stutio v8.0.0.843, San Diego, CA, USA). The detailed schemes and parameters adopted in DMol3 calculations are listed in Table 1.

## 3. Results and Discussion

### 3.1. Material Characterization

Thermal elongation and gel extraction experiments are carried out by referring to the standards of GB/T 2951.21-2008 and ASTMD 2765-2011, respectively. It is explicit that the XLPE crosslinking degrees being initiated by UV irradiation with TMPTMA have reached an adequate 25% in thermal elongation to be mechanically qualified for cable productions, implying the competent efficiency of UV-initiation crosslinking technique with TMPTMA as the auxiliary crosslinking agent. 

**Figure 2:**
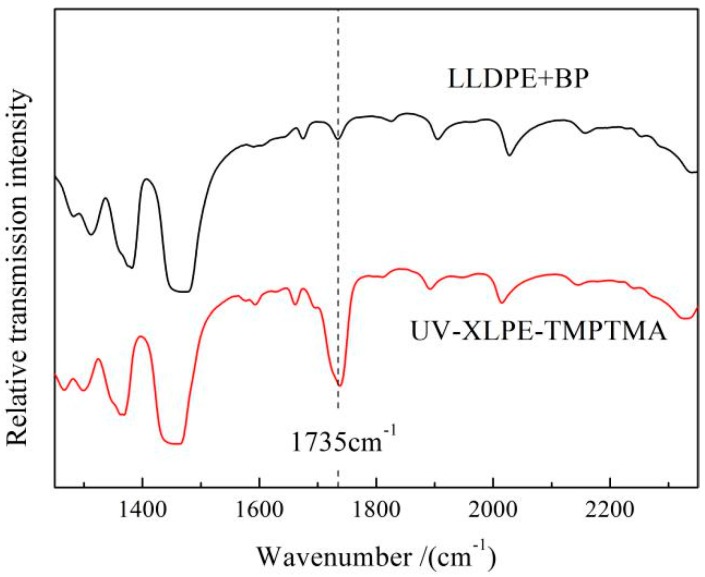
Infrared transmission spectra of LLDPE + BP mixture and UV-XLPE-TMPTMA.

The molecular structure of UV-XLPE-TMPTMA is characterized by IR spectroscopy in comparison to the homologous blend sample obtained though the similar preparation process without TMPTMA (LLDPE + BP), as the results shown in Figure 2, indicating the molecular structural variation originating from UV-initiated crosslinking reactions. The TMPTMA can be identified by the stretching vibration peaks of carbonyl (C=O) at 1735 cm^−1^ in IR transmission spectra, which are engendered by UV irradiation and retain after hot-degassing treatment. Especially for the mixture of LLDPE and BP without auxiliary crosslinking agent, this characteristic peak does not arise even through UV irradiation process, confirming the definite correlation of this characteristic peak with the auxiliary crosslinker being chemically connected (grafted) between polyethylene molecules. The FT-IR results reasonably prove that TMPTMA have been successfully grafted to polyethylene molecules.

### 3.2. Space-Charge Characteristics

**Figure 3:**
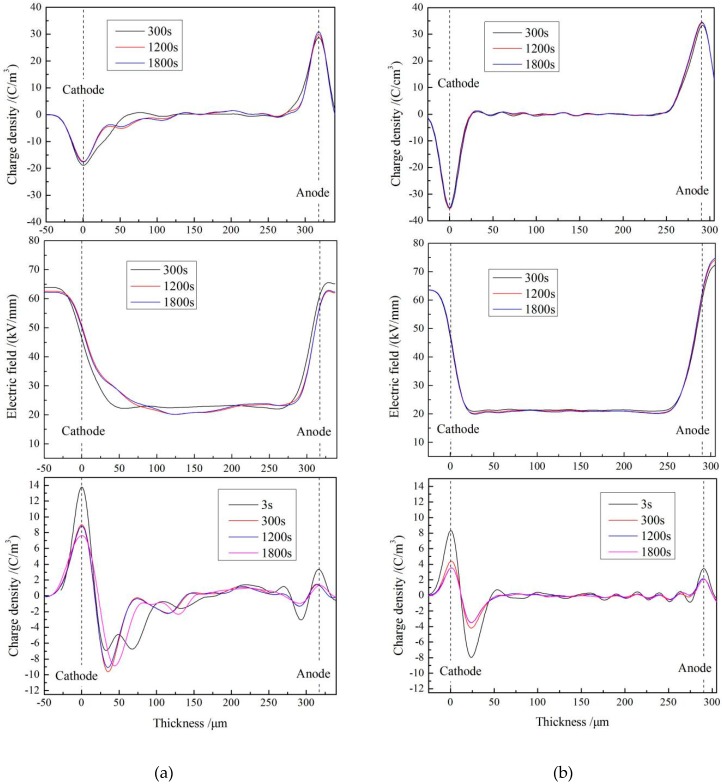
Space-charge distributions at 25 °C in (**a**) LLDPE, (**b**) UV-XLPE-TMPTMA under applied DC electric field 40 kV/mm (upper panels) and in short circuit (bottom panels). The electric field distributions across samples in polarization process are illustrated in middle panels.

Space-charge characteristics of LLDPE and UV-XLPE-TMPTMA are tested with pulsed electro-acoustic (PEA) method to reveal the underlying behavior of charge injections. Figure 3 illustrates the space-charge accumulation under the applied DC electric field of 40 kV/mm (upper panels) and the short-circuit dissipation of accumulated space charges (bottom panels) for 30 min at a temperature of 25 °C. It is indicated from the upper panel of Figure 3a that plenty of homocharges have been evidently accumulated near the cathode, and the charge density increases with polarization time to the highest value of 4 C/m^3^, implying the migration of negative space charges into LLDPE under the DC electric field. Meanwhile, a few homocharges are also injected into the surface of the LLDPE samples from the anode. In comparison, the crosslinked sample UV-XLPE-TMPTMA represents a gentle varying curve of space-charge distributions even after being polarized for 1800 s, but with a small amount of heterocharges accumulating near cathode with a peak value of 1.5 C/m^3^. The minimal quantity of positive space charges distributing inside UV-XLPE-TMPTMA and near the anode further demonstrates that the depth of space-charge injection and the density of space-charge accumulation can be substantially reduced by UV-initiated crosslinking with a TMPTMA auxiliary agent.

Electric field distributions across samples in the polarization process are shown by the variation curves in the middle panels of Figure 3. Although the electric field distribution in LLDPE is stable before 300 s, the intensively fluctuating curves of 1200 s and 1800 s consistently indicate that space-charge accumulations in the polarization process have approached saturation with a considerable distortion of internal electric field. By contrast, the electric field distributions in UV-XLPE-TMPTMA at 300 s and 1200 s are almost identical with a more stable variation than LLDPE, which demonstrates that the uniformly distributed electric field has not been distorted by space-charge accumulations despite the complete polarization process.

Space-charge characteristics of LLDPE and UV-XLPE-TMPTMA in short-circuit process are exhibited in the bottom panels of Figure 3. The peak density value of short-circuit charges in LLDPE reaches a relatively larger value of 9 C/m^3^. Many homocharges are observed to appear at cathode with a peak density of 1.5 C/m^3^ while a few heterocharges emerging at anode, with the accumulated charges being dissipated to a convergent state after short-circuiting for an adequately long time of 1200 s. Nevertheless, the peak value of charge density in UV-XLPE-TMPTMA is appreciably decreased to 3.5 C/m^3^ with a faster dissipating rate of short-circuit process, in which residual charge distribution remains unchanged after 300 s. Therefore, it can be reasonably suggested that the higher ability of suppressing space-charge accumulation has been acquired by UV-XLPE-TMPTMA, which is attributed to the deep traps deriving from the polar group of TMPTMA, as elucidated by the following TSDC analyses and first-principles calculations.

### 3.3. Electric Charge Traps

The TSDC testing temperature range of −50–150 °C covers the thermal excitation energies of the charges captured by both intrinsic traps of XLPE and TMPTMA-introduced deep traps, in which the practical operating temperatures of high voltage DC cable are completely included. TSDC test characterizes the current produced by the thermally excited charges which have been trapped by bound states of electron or holes, in which the intensity and position of current peak signify the whole quantity of the trapped charges throughout the material and the trapping level depth respectively [21]. Accordingly, a new peak in TSDC spectra of XLPE-TMPTMA arising at a higher temperature identifies a deeper tapping level, while the lower temperature peak corresponds to the intrinsic traps from structural defects of polyethylene materials.

**Figure 4:**
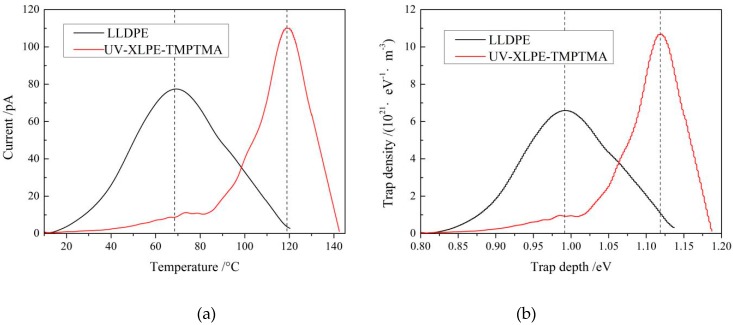
(**a**) Temperature spectra of thermally stimulated depolarization currents and (**b**) the trap level distributions of LLDPE and UV-XLPE-TMPTMA.

It is illustrated from the measured TSDC spectra and the derived trap level distributions of LLDPE and UV-XLPE-TMPTMA as shown in Figure 4 that a lot of deeper traps have been introduced by TMPTMA crosslinker. The deepest trap level peaks of LLDPE and UV-XLPE-TMPTMA are 0.99 and 1.12 eV with trap level densities of 6.61 and 10.76 (10^21^ eV^−1^·m^−3^) respectively. The homogeneously distributed deep traps fixed on the grafted TMPTMA will effectively capture the injected hetero-charges and thus form intensified electrostatic shielding layers near electrodes under applied DC electric field, which can eventually block charges being further injected from electrodes and impede carrier transport. Consequently, the space-charge accumulation in UV-XLPE-TMPTMA is remarkably suppressed due to the deep traps introduced by the TMPTMA connecting polyethylene molecules. Furthermore, auxiliary-introduced traps, being 0.13 eV deeper than intrinsic traps, can retain the highly efficient suppression mechanism of space accumulation at a temperature approaching to 118 °C, which is significantly higher than the 68 °C for LLDPE without auxiliary crosslinking. These results suggest that UV-XLPE-TMPTMA can persist in high DC dielectric performance when the cable working temperature increases to 118 °C.

**Figure 5:**
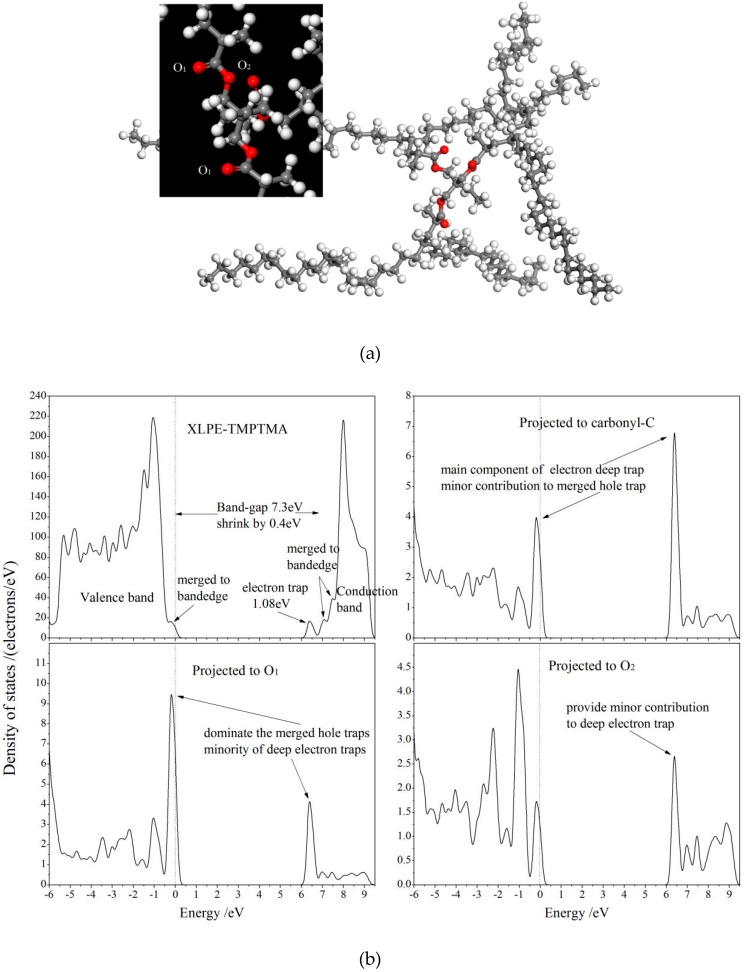
(**a**) Schematic molecular structure (inset magnifies TMPTMA crosslinker) and (**b**) total and atomic-projected density of states for XLPE-TMPTMA molecule in which the highest occupied molecular orbital is referenced as energetic zero being indicated by vertical dashed lines.

To establish a theoretical basis of accounting for the improved DC dielectric performance of UV-XLPE-TMPTMA, the first-principles electronic structure calculations are performed for the multiple polyethylene molecules being chemically connected by an auxiliary crosslinker (XLPE-TMPTMA), as the representative structural schematics of the relaxed molecule and the energetic distribution of electronic states (total and atomic-projected density of states), as shown in Figure 5. The grafted TMPTMA will introduce three unoccupied electronic bound states in the band-gap of polyethylene molecules, one of which can serve as the deep traps of free electrons with the energy level of 1.08 eV below conduction band minimum (CBM), which agrees well with experimental values of 1.12 eV from TSDC tests. Very close to CBM and valence band maximum (VBM) of polyethylene, two unoccupied bound states and one occupied bound state are also respectively introduced by the TMPTMA crosslinker, all of which have merged into the band edge to become a new CBM and VBM. The projected density of states (Figure 4b) shows that the deep trap states of 1.08 eV are mainly contributed by the atomic orbitals of carbonyl–carbon atom (>70%) with a small fraction (<30%) deriving from the carbonyl–oxygen (O1) and ester oxygen (O2) of the grafted TMPTMA, where the electron wave functions are distributed in local space around the carbonyl bond (C=O). The first-principles electronic structures, being consistent with experimental results of TSDC, further prove that the polar carbonyl group can introduce electronic states of deep traps with energy levels among the band-gap of polyethylene molecules, which can efficiently scatter and capture electrons in the conduction band. These theoretical results elucidate the underlying molecular physics of improving space-charge distributions in XLPE by grafting auxiliary crosslinking agent in UV-initiated crosslinking process.

### 3.4. Electrical Conductance

**Figure 6:**
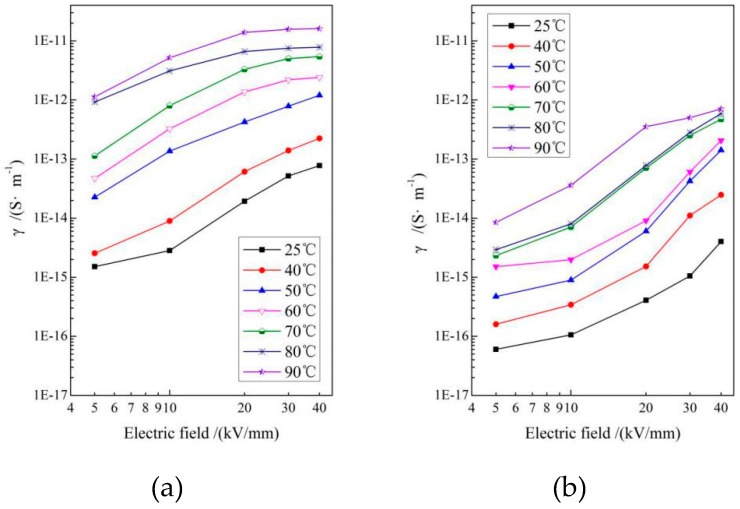
*γ*-*E* curves of (**a**) LLDPE and (**b**) UV-XLPE-TMPTMA at various temperatures of 25–90 °C.

The electrical conductance of XLPE materials is highly dependent on the temperature and electric field, as the conductivity-electric field (*γ*-*E*) varies with the archetypal operating temperature of DC insulated cable (25–90 °C) shown in Figure 6. The conductivity of UV-XLPE-TMPTMA is appreciably lower than LLDPE for all the testing temperatures. The *γ*-*E* curves also illustrate the evident increment of critical electric field caused by grafting auxiliary crosslinking agents onto polyethylene molecules. The density of states near the CBM and VBM of the polymer molecules directly determines the probability of carrier transition between the adjacent levels of electronic states at the band edge, and thus dominates the carrier mobility without consideration of impurity or defect scattering. As illustrated in Figure 5b, a considerable amount of bound states have been introduced near the band edge by grafting TMPTMA, leading to substantially lower density of states at both CBM and VBM, which implies lower carrier mobility and thus consistently explains the considerably decreased conductivity of UV-XLPE-TMPTMA. Based on electronic structure theory of condensed matter physics, carrier mobility can be reduced from carrier-phonon scattering only when the temperature is highly raised, so we can eliminate the temperature effect on carrier mobility of polyethylene materials at 25–90 °C. Therefore, the conductivity of polyethylene molecules being crosslinked by TMPTMA auxiliary crosslinker is invariably lower than LLDPE at the temperature range for cable working of 25–90 °C.

## 4. Conclusions

Exploiting auxiliary crosslinking agents in the photon-initiated crosslinking process, the DC dielectric performance of XLPE has been evidently improved with UV irradiation technology. The underlying mechanism of suppressing charge injection and restraining electrical conductance is elucidated by TSDC analysis and quantum mechanics calculations in combination with the conductivity field variation at various temperatures of cable operation. The auxiliary crosslinking efficiency of UV-XLPE-TMPTMA has been verified by thermal elongation test and IR transmission spectra. It has been demonstrated that UV irradiation technique can successfully initiate crosslinking reactions between polyethylene molecules and auxiliary crosslinking agents to achieve modified XLPE with sufficient crosslinking degree and excellent thermal elongation performance. The modified DC dielectric performance of UV-XLPE-TMPTMA is attributed to the deep traps engendered by the polar carbonyl of grafted TMPTMA which is testified by TSDC analysis and first-principles calculations. The charges, being captured by these deep traps in higher density, will form effective electrostatic shielding layers near electrodes to impede charge injections and thus remarkably inhibit space-charge accumulation inside XLPE materials. Compared with LLDPE, lower conductivity has been acquired by the XLPE with TMPTMA as an auxiliary crosslinker which represents prominent efficiency of ameliorating dielectric properties. The first-principles calculations also indicate that the multiple electronic bound states can be engendered by the grafted TMPTMA, which will decrease the density of state at the edges of both conduction and valance bands, consequently leading to decreased carrier mobility. The present study suggests a prospective strategic scheme of UV-initiated graft modification for realizing high-performance insulating materials in HVDC cable production. Nevertheless, this paper only presents the most important study on the DC dielectric performance of modified XLPE, concerning the improvement of space-charge accumulation and the suppression of electrical conductance. For practical applications in DC insulated cable, the other electrical properties, such as DC breakdown strength when the polarity reverses, and electric tree characteristics under impulse voltage or DC short circuit, should be further researched in future work.

## Figures and Tables

**Table 1:** Schemes and parameters adopted in the first-principles calculations by DMol3.

Electronic Hamiltonian	Scheme	Condition and Parameter
Exchange-correlation energy	Meta-generalized-gradient approximation	M11-L [20]
Integration accuracy		2000 grid points /atom
SCF	Tolerance	1×10^−6^ eV/atom
	Multipolar expansion	Octupole
	Charge density mixing	Charge = 0.3, DIIS = 5
Core treatment	All-Electron	
Numerical basis set	DNP	Basis file 4.4
Orbital cutoff	Global	5.0 Å
